# Supplementary material for: SyNDI: synchronous network data integration framework
Source: BMC Bioinformatics. 2018 Nov 6;19:403. doi: 10.1186/s12859-018-2426-5 (PMC6219086; doi:10.1186/s12859-018-2426-5)
Supplement: Supplementary file 5 — Files of the example presented in Additional file 4. This additional file is a zip package that contains all files used in Additional file 4. Detailed descriptions of each of these files are presented in a README file included in this zip package. (GZ 30411 kb) [file 12859_2018_2426_MOESM5_ESM.gz › SyNDITools/Meme2Fimo/meme_4.9.0_4.tar.gz!/meme_4.9.0/doc/glam2format_man.html]

glam2format Manual


# glam2format Manual

This program reads in a motif found by glam2, and writes it in a
standard alignment format (FASTA-with-gaps or MSF). This enables the
alignment to be passed to third-party software, including graphical
visualization tools such as Kalignvu,
Boxshade,
and WebLogo. On the other
hand, not all the motif information is preserved: in particular, the key
positions are lost. Only the top motif in glam2 output is converted.

- Basic usage:

  ```
  glam2format fasta my_motif.glam2
  ```

  ```
  glam2format msf my_motif.glam2
  ```
- Use -o to write the output to a file rather than to the
  screen:

  ```
  glam2format -o my_motif.fa fasta my_motif.glam2
  ```

  ```
  glam2format -o my_motif.msf msf my_motif.glam2
  ```
- Use -c to make a compact alignment:

  ```
  glam2format -c fasta my_motif.glam2
  ```

  ```
  glam2format -c msf my_motif.glam2
  ```

  By default, residues that are inserted between key positions are
  written as unaligned with each other. This best reflects glam2's
  intention, but it can make the alignment large and full of gaps. With
  -c, inserted residues are written as arbitrarily aligned with each
  other, just as they appear in the glam2 output.
- Use -f to make a 'global' alignment by adding flanking
  sequences from the original FASTA-format sequence file:

  ```
  glam2format -f my_seqs.fa fasta my_motif.glam2
  ```

  ```
  glam2format -f my_seqs.fa msf my_motif.glam2
  ```

  The flanking sequences will be written as either unaligned with each
  other or arbitrarily aligned, depending on the -c option. The sequences
  should have unique names and their order should be unchanged.
